# Systematic organisation of skinfold callipers: an approach based on physical-mechanical properties and characteristics

**DOI:** 10.1017/S0007114525105473

**Published:** 2025-12-14

**Authors:** Joaquim Cintra, Timothy Lohman, Francesco Campa, Steven Heymsfield

**Affiliations:** 1 Federal University of Ceará, Fortaleza, CE, Brazil; 2 Emeritus Faculty, University of Arizona, Tucson, AZ, USA; 3 Department of Biomedical Sciences, University of Padua, Padua, PD, Italy; 4 Pennington Biomedical Research Center, Baton Rouge, LA, USA

**Keywords:** Anthropometry, Body composition, Nutritional assessment, Skinfold thickness, Skinfold calliper

## Abstract

Skinfold callipers are used internationally in research, clinical and field settings to assess body composition and nutritional status. Notably, currently available instruments differ in important specificities that impact measurement. In this sense, this report proposes a methodological approach that organises skinfold callipers into three categories (*Original*, *Generic* and *Hybrid*) and three configurations (*Type A*, *Type B* and *Type C*) based on physical-mechanical properties and characteristics. Therefore, this concept provides technical support for choosing the most appropriate skinfold calliper in different contexts.

Skinfolds represent an anthropometric-specific property used to describe body composition according to the 5th level of organisation: the whole-body level^(1)^. They are derived from measurements taken on the individual’s body surface, which determine the thickness of a double layer consisting of skin and subcutaneous adipose tissue at defined anatomical sites^(2)^. These measurements can then be used in a qualitative approach to assess body composition and monitored longitudinally as indicators of variations in body fat, as they are strongly associated with parameters related to health and athletic performance^(3)^. Alternatively, they can be used in mathematical models to quantify components belonging to other levels of organisation of body composition, such as the molecular (2nd) or tissue system (4th) level, thus estimating fat mass or adipose tissue mass, respectively^(4,5)^.

Although alternative methods, such as ultrasound, have been explored to assess skinfold thickness, it remains a strictly anthropometric measure that can only be obtained with callipers. These instruments compress the skinfolds with a standardised mechanical pressure, equivalent to that applied when pinching with the thumb and index finger^(6)^. Callipers serve as a support for the operator’s hand, since although they can form the skinfold, they cannot quantify it^(7)^. Over the years, numerous skinfold callipers have been developed and employed in the literature, yet no study has systematically organised them based on their defining features.

In contrast, other methods for assessing body composition classify their instruments into specific categories. For example, in bioelectrical impedance analysis, devices are grouped either by technology (hand-to-hand, leg-to-leg, foot-to-hand and direct segmental) or by frequency (single-frequency and multifrequency)^(8)^. Establishing similar classifications for skinfold callipers is important, particularly in light of advances in both conventional and digital anthropometry and the ongoing evolution of calliper designs. Identifying key physical and mechanical features enables the evaluation of potential similarities and differences among instruments. Therefore, this report aims to systematically organise skinfold callipers into categories and configurations based on their physical-mechanical properties and characteristics.

## Development

**Fig. 1.**
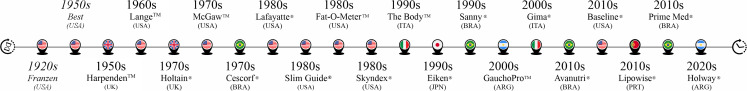
Callipers historically used to measure skinfold thickness (1920s–2020s).

The skinfold calliper is a specialised anthropometric instrument used to measure skinfold thickness. Notably, more than twenty callipers have been proposed over a 100-year journey of advancements in skinfold assessment and human body composition (Fig. 1)^(9–11)^. These instruments were developed by manufacturers in Asia, Europe, Latin America and North America. Early models were structurally rudimentary and were discontinued in the 1950s, after James Mourilyan Tanner (1920–2010) introduced a prototype calliper optimised with *helical extension spring* kinematics in 1953, which has since been considered the defining mechanical feature of a skinfold calliper^(9)^. Conversely, callipers that do not incorporate this principle are limited to a conventional precision instrument for measuring rigid, opposing surfaces. Therefore, the classic study by Edwards *et al.* ^(12)^, published in the *British Journal of Nutrition* in 1955, represents a milestone in the theoretical foundation of skinfold callipers.

### Skinfold callipers: systematic organisation by category and configuration

For decades, skinfold callipers have been classified according to their application settings: *clinical* or *scientific*. However, this approach, which originated in Brazil, is unfounded, influenced by commercial interests, and, most importantly, disregards critical physical-mechanical properties and characteristics^(9)^. In this report, we propose the first systematic organisation of skinfold callipers based on these attributes. Thus, in our methodological framework, *properties* refer to the structural components present in all skinfold callipers, such as the jaws, springs and dial, whereas *characteristics* describe measurable aspects associated with these properties, such as jaw surface area, spring force and dial type and resolution. Consequently, skinfold callipers can now be organised into three categories: *Original*, *Generic* and *Hybrid*. These categories will be detailed in the following sections.

#### Original skinfold callipers: the reference instruments

**Fig. 2.**
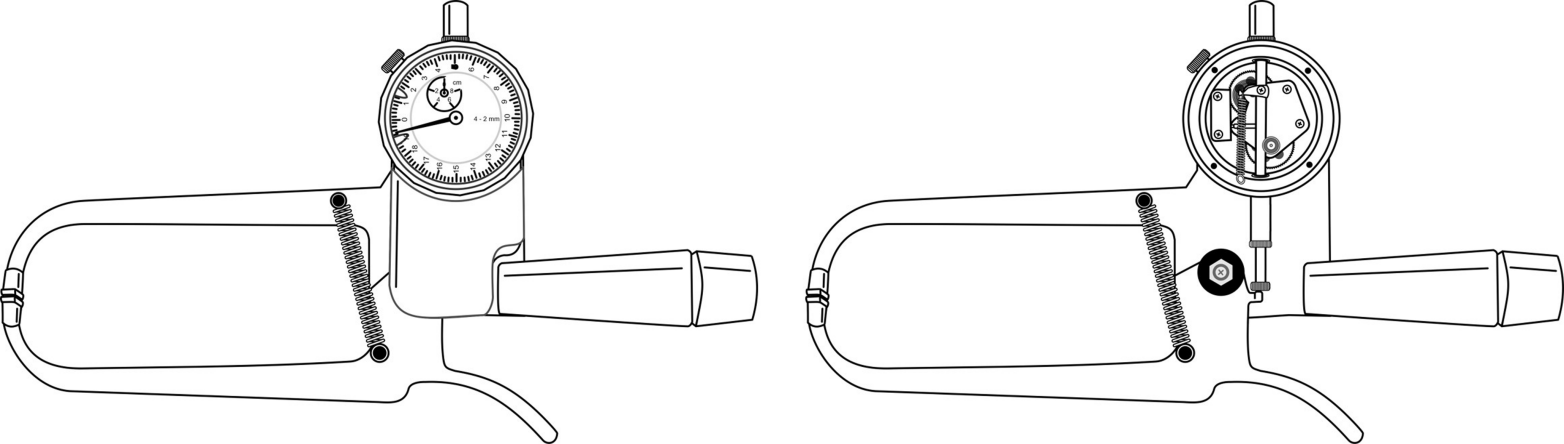
Original *Type A* skinfold calliper: *The Harpenden* ^*™*^.

The original skinfold callipers exhibit a specific physical-mechanical configuration based on a set of well-defined structural properties and functional characteristics, which constitute a reference standard. These key parameters include the lever class, jaw surface area, spring attachment point and angle, downscale force and pressure and dial type and/or resolution, among others. In 2023, the *Harpenden* ^™^ (Baty International), *Lange* ^™^ (Beta Technology) and *Slim Guide* ^®^ (Creative Health Products) skinfold callipers were designated as reference models, establishing the three configurations: *Type A*, *Type B* and *Type C*, respectively^(9)^. These are presented below:

Original *Type A* skinfold calliper (Fig. 2): Designed with a third-class lever, the physical structure and mechanical components are metal. The jaws are rectangular with a surface area of 90 mm^2^ (6 × 15 mm). Two extension springs are installed parallel and obliquely on the sides of the rods and in front of the pivot pin. The dial is an analogue indicator with a resolution of 0·2 mm and a range of 0–80 mm. The mean static downscale force and pressure are 743 ± 12·9 g and 8·25 ± 0·3 g/mm², respectively, at 10–50 mm intervals^(9,11)^.

**Fig. 3.**
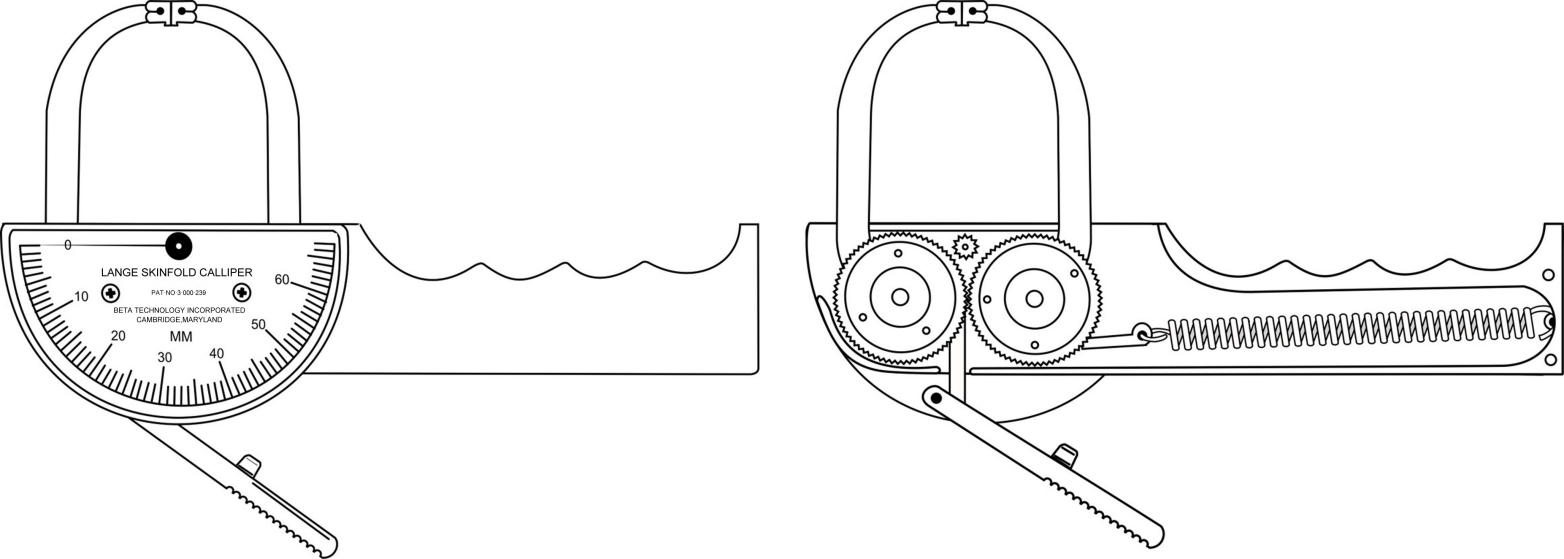
Original *Type B* skinfold calliper: *The Lange* ^*™*^.

Original *Type B* skinfold calliper (Fig. 3): Designed with a first-class lever, the physical structure and mechanical components are metal. The jaws are rectangular with a surface area of 30 mm² (5 mm × 6 mm). A single extension spring is installed transversely to the handle and a rod that connects to the trigger-driven gears. The dial is a semicircular analogue scale with 1·0 mm resolution and a range of 0–60 mm. The mean static downscale force and pressure are 250 ± 6·3 g and 8·37 ± 0·2 g/mm², respectively, at 10–50 mm intervals^(9,11)^.

**Fig. 4.**
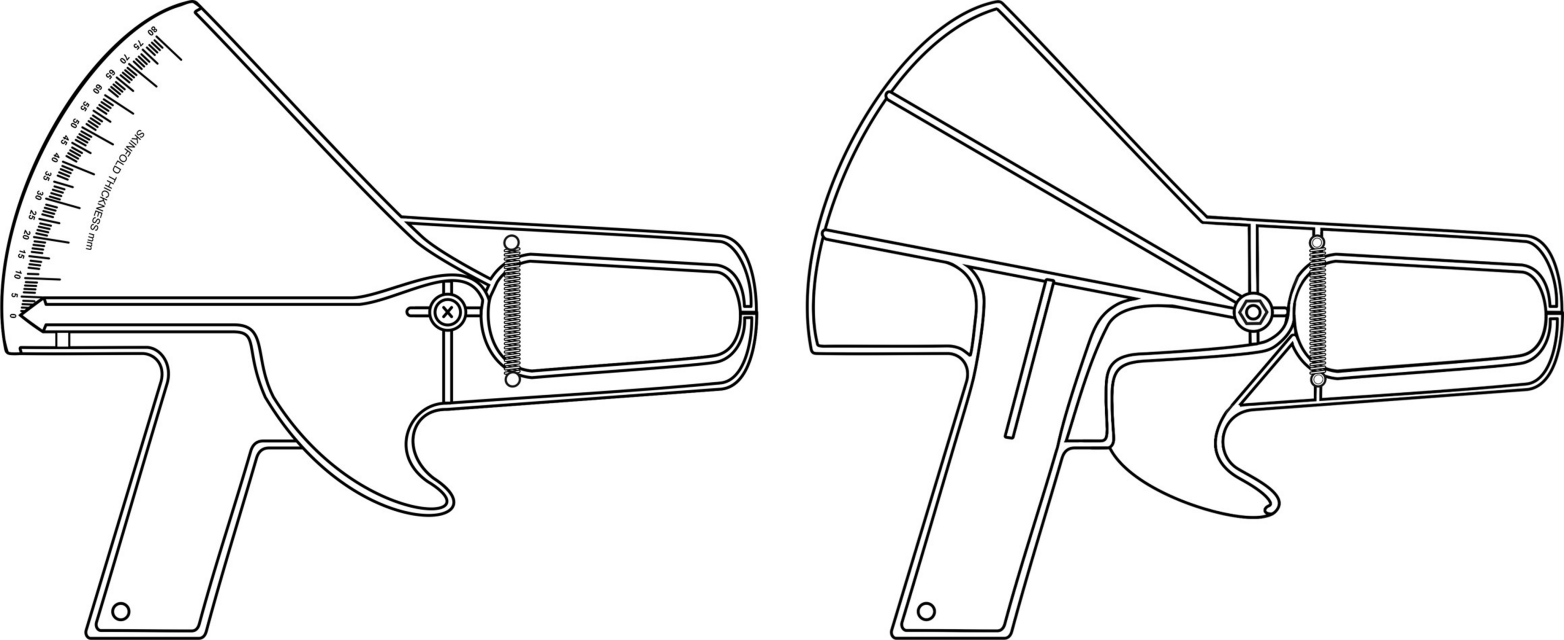
Original *Type C* skinfold calliper: *The Slim Guide* ^*®*^.

Original *Type C* skinfold calliper (Fig. 4): Designed with a third-class lever, the physical structure is plastic, and the mechanical components are metal. The jaws are rectangular with a surface area of 91 mm² (7 mm × 13 mm). Two extension springs are installed parallel and vertically on the sides of the rods and in front of the pivot pin. The dial is an analogue linear scale with 1·0 mm resolution and a range of 0–80 mm. The mean static downscale force and pressure are 683 ± 23·7 g and 7·51 ± 0·3 g/mm², respectively, at 10–50 mm intervals^(9,11)^. Additional information about the original skinfold callipers is presented in Table 1.

**Table 1.** Original, generic and hybrid skinfold callipers described according to the new systematic organisation

	Country	Name	Model	Material	Type	Lever	Jaws (mm²)	Pressure (g/mm²)	Force (g)	Dial	Resolution (mm)	Range (mm)	Weight (g)
Original	UK	Harpenden^™^	N/A	Metal	A	3rd Class	90	8·2	742	Indicator	0·2	0–80	498
	USA	Lange^™^	N/A	Metal	B	1st Class	30	8·4	251	Scale	1·0	0–60	200
	USA	Slim Guide^®^	N/A	Plastic	C	3rd Class	91	7·5	683	Scale	1·0	0–80	122
Generic	UK	Holtain^®^	N/A	Metal	A	N/R	N/R	10·0*	N/R	Indicator	0·2	0–46	400
	USA	Skyndex^®^	N/A	Plastic	A	3rd Class	102	7·3	744	Electronic	0·1	0–60	398
	BRA	Cescorf^®^	Clinical	Metal	A	N/R	N/R	10·0*	N/R	Scale	1·0	0–75	190
	BRA	Sanny^®^	AD1007	Metal	A	N/R	N/R	9·8*	N/R	Indicator	0·1	0–70	N/R
	BRA	Avanutri^®^	Scientific	Metal	A	N/R	N/R	10·0*	N/R	Indicator	0·1	0–83	388
	BRA	Prime Med^®^	A30	Metal	A	N/R	N/R	9·8*	N/R	Indicator	0·1	0–92	N/R
	ITA	Gima^®^	27 320	Metal	A	N/R	N/R	10·0*	N/R	Indicator	0·1	0–40	N/R
	ARG	Holway^®^	N/A	Metal	A	N/R	N/R	10·0*	N/R	Scale	1·0	0–60	168
	USA	Lafayette^®^	01127A	Metal	B	1st Class	30	7·5	225	Scale	1·0	0–100	317
	USA	Baseline^®^	12–1110	Metal	B	N/R	N/R	N/R	N/R	Scale	1·0	0–70	N/R
	BRA	Cescorf^®^	Innovare	Plastic	C	N/R	N/R	10·0*	N/R	Scale	1·0	0–80	95
	BRA	Avanutri^®^	Clinical	Plastic	C	N/R	N/R	10·0*	N/R	Scale	1·0	0–80	80
Hybrid	PRT	Lipowise^®^	Pro	Metal	N/A	N/R	N/R	10·0*	N/R	Electronic	0·1	0–100	260

BRA, Brazil; ITA, Italy; PRT, Portugal.

Note: Pressure and force: static downscale. *Static upscale pressure. N/A: not attributed. N/R: Not reported by the manufacturer or in the literature. The Lafayette^®^ calliper was discontinued in 2004.

Since the 1970s, the physical-mechanical configurations of the Harpenden^®^ and Lange^™^ skinfold callipers, proposed by Edwards *et al.* ^(12)^ in 1955 and Lange and Brozek^(13)^ in 1961, respectively, have been widely adopted internationally as the main reference standards for the development of new skinfold callipers^(9)^. Consequently, the generic and hybrid categories, corresponding to equivalent and combined variants of these instruments, constitute an expansion of the *original category*, as presented and described below:

#### Generic skinfold callipers: the equivalent instruments

The generic skinfold callipers have a typical physical-mechanical configuration based on an original skinfold calliper, such as Holway^®^ (Holway Anthropometric Equipment), Lafayette^®^ (01127A, Lafayette Instrument Company) and Cescorf^®^ (Innovare-4^™^, Cescorf Equipment), which can now be classified as *Type A*, *Type B* and *Type C* generic skinfold callipers, respectively. The term *generic* does not imply inferior quality, but rather callipers that have properties and characteristics identical or equivalent to their original counterparts. However, potential differences in performance or compliance were not explored in this scientific report, as they are beyond its scope, which is limited to the organisation of skinfold callipers. Furthermore, for commercial regulatory contexts, this analysis should be systematically evaluated by federal agencies specialising in metrology, such as the US *National Institute of Standards and Technology*, among others.

Some generic skinfold callipers within the same configuration, such as Holtain^®^ (Holtain), although mechanically similar, may present structural inconsistencies compared with the original skinfold callipers. Thus, researchers have suggested that these differences are primarily due to physical factors, such as the spring attachment point and angle and the jaw surface area, as well as aspects related to the quality, condition and integrity of the pivot components (e.g. *screw*, *washer* or *gear*) and the calibration procedures employed by the manufacturers^(9,11)^. Comparative studies indicate that, in some generic callipers, such structural deviations do not appear to significantly compromise functional performance. Lohman *et al.* ^(14)^ demonstrated high inter-operator agreement using skinfold callipers with the same physical-mechanical configuration. Schmidt and Carter^(11)^ and Esparza-Ros *et al.* ^(15)^ reported that some original and generic skinfold callipers provided statistically equivalent skinfold measurements. However, skinfold callipers cannot be used interchangeably to measure skinfold thickness and subsequently assess body adiposity^(9,11,14,15)^.

The generic skinfold callipers manufactured by Cescorf^®^ have received international recognition^(16)^. Significant improvements in mechanical performance, especially in the *Type A* models, were groundbreaking. The pivot components are now made of polyacetal to reduce the coefficient of friction, thus allowing more elastic energy to be available in the two springs during the downscale actions^(9)^. In addition, because the two metal rods are connected in parallel by the pivot and are not convergent, the upper fixed rod now has a slight sinuosity that, according to the manufacturer, allows the jaws to align harmoniously. Another *Type A* model also features movable jaws that better adapt to the skinfold, while some have been optimised with a linear scale, replacing the analogue dial indicator. In this context, based on the 1979 study by Jones *et al.* ^(17)^, an improved generic *Type A* skinfold calliper with a digital dial indicator was introduced by Cescorf^®^ in 1985, pioneering this development in Latin America. However, due to import restrictions on this component, production was later discontinued and only resumed in 2016. The ease of reading the measurement represents a notable strength of the device. Despite this, the reliability and cost-effectiveness of this calliper are questionable, given the dial indicator’s susceptibility to impacts and the frequent need for calibration. Although this procedure can be performed by the operator using the *Gauge-Block* provided in the case, in most situations, it still requires manufacturer intervention, resulting in additional shipping costs^(9)^. In 2025, an updated version of this skinfold calliper was introduced, incorporating an improved digital dial indicator, which, according to the manufacturer, offers greater metrological stability. Finally, the generic *Type C* skinfold calliper from the same manufacturer has been progressively optimised in four versions over the past 15 years. Notably, its structural dimensions have been ergonomically compacted, and the spring attachment angle and jaw area have been reduced. Therefore, static and dynamic calibration studies, predominantly based on load cells, among other reference metrological methods, should be conducted on all these generic skinfold callipers to assess the effectiveness and practical implications of the aforementioned improvements in skinfold thickness measurement.

#### Hybrid skinfold callipers: the combined instruments

The hybrid skinfold callipers have an atypical physical-mechanical configuration based on two original skinfold callipers. The Lipowise^®^ (Wisify Tech) represents the first generation of skinfold callipers developed by integrating the key physical and mechanical characteristics of the *Type A* and *Type B* configurations, such as the jaw surface area and force transmission system, respectively^(9)^. Notably, the crucial difference lies in how the spring force is kinematically transmitted and applied: Lipowise^®^ converts the spring force into torque through a lever shaft on the same rod, while Lange^®^ applies the force directly and symmetrically through a 1:1 gear system that connects the rods. Therefore, given its hybrid nature, no typical configuration (Type A, Type B or Type C) can be attributed to instruments in this category. Furthermore, the Lipowise^®^ calliper incorporates technological innovations, including digital measurement automation linked to a smartphone app via *Bluetooth* ^(9)^. Finally, similar improvements are being introduced in other anthropometric instruments, such as ultrasonic stadiometers. Recently, Brazilian researchers validated a portable device developed in South Korea to measure standing stature in adults^(18)^.

Absolute differences between the original and hybrid skinfold callipers have recently been documented^(15,19)^. Esparza-Ros *et al.* ^(15)^ demonstrated that the Lipowise^®^ calliper provided skinfold measurements at eight sites that were statistically equivalent to those obtained with the Harpenden^®^ calliper. Similarly, Leão *et al.* ^(19)^ reported no significant differences between these instruments. However, no studies have directly compared the Lipowise^®^ and Lange^™^ callipers. Furthermore, although the Lipowise^®^ incorporates features of both *Type A* and *Type B* configurations, the available evidence is limited to comparisons with the Harpenden^®^, so it is not yet possible to precisely determine which configuration most closely matches its functional performance. Future studies should address this issue.

**Fig. 5.**
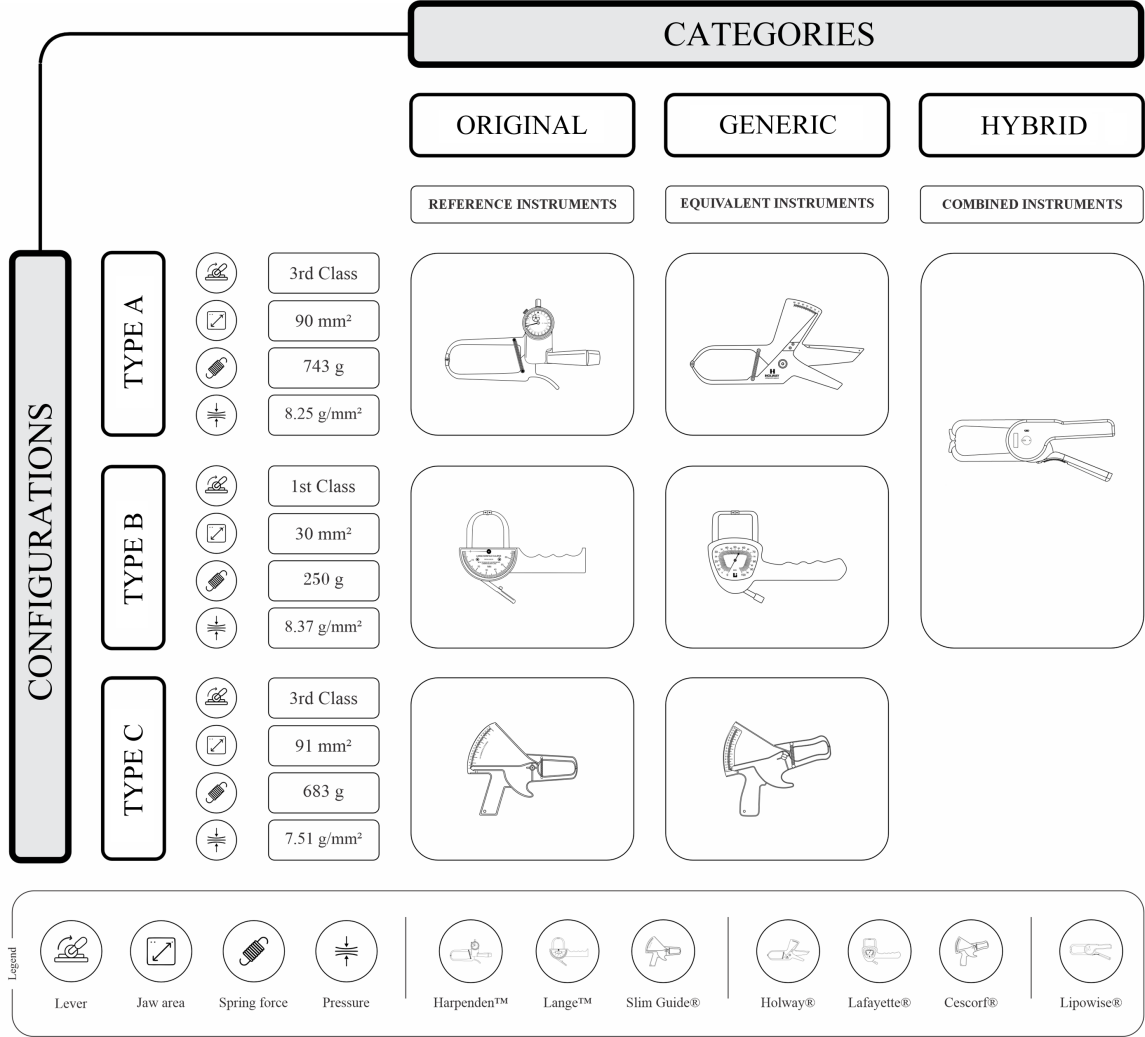
Organisation of skinfold callipers by category and configuration.

The organisational structure of skinfold callipers into categories and configurations provides a comprehensive approach that consolidates the instruments into a single, coherent classification. Figure 5 schematically illustrates this paradigm based on the critical physical-mechanical characteristics of the original models, including lever class, jaw surface area, spring force and static downscale pressure. Notably, although the generic skinfold callipers presented in Fig. 5 were selected by the author for convenience, their inclusion was determined by objective attributes rather than historical or commercial considerations.

### Skinfold callipers: instrumental description and incremental evidence

The most commonly used skinfold callipers in research, clinical and field settings were described and systematically organised into categories and configurations based on their physical and mechanical properties and characteristics (Table 1). Instruments recognised by international groups specialising in anthropometry and body composition, such as the *International Society for the Advancement of Kinanthropometry* and the *Global Institute for Health and Body Composition*, respectively, were included, as well as those used in studies that characterised population anthropometric profiles or proposed predictive regression equations based on skinfold thickness. Furthermore, criteria such as commercialisation and adoption in various geographic and socio-economic contexts were also considered, with priority given to skinfold callipers with the largest market share in developing and developed countries, such as Brazil and the USA, respectively. Finally, an observational and comparative analysis was also conducted using the original skinfold callipers as a reference to categorise the remaining callipers as generic or hybrid.

Sixteen skinfold callipers were described in Table 1: three original, twelve generic and one hybrid. Brazil and the USA lead industrial production. Some manufacturers have introduced multiple generic models within the same configuration. This variety is notable in Brazilian product lines such as Avanutri^®^, Cescorf^®^, Prime Med^®^ and Sanny^®^. However, these additional instruments were not included in this report. Most skinfold callipers are metallic, relatively lightweight, and feature a semicircular or linear scale dial with a 1·0 mm resolution (Table 1). Furthermore, most commercially available generic skinfold callipers are predominantly based on the *Type A* configuration. Finally, driven primarily by international accreditation courses in anthropometry from the International Society for the Advancement of Kinanthropometry, which currently has members in eighty-five countries, both original and generic *Type C* skinfold callipers are frequently used in clinical settings^(9)^.

The jaw surface area and spring force were reported as the main physical and mechanical characteristics for selecting a skinfold calliper, since the upscale pressure of 10 g/mm² and the downscale pressure of 8 g/mm² may be confounding factors, as they are obtained with different combinations of *force* (g) and *area* (mm²)^(9)^. However, no manufacturer publicly discloses these technical specifications. We contacted customer service for more details. Those who responded to our inquiries cited unavailability or confidentiality as reasons for not disclosing the information. Consequently, only 31 % of the instruments were fully described (Table 1). Indeed, although these variables could have been determined through our own analyses, we emphasise that this gap highlights a substantial deficiency in the availability of technical information provided by manufacturers. Furthermore, calibration studies are scarce in the specialised literature and are limited to a few skinfold callipers^(11)^.

Notably, over the last century, the skinfold measurement technique has been extensively explored and has well-defined standards^(2,7)^. Therefore, the construction, calibration and maintenance of skinfold callipers must be standardised and regulated internationally based on the category and physical-mechanical configuration. To this end, the technical manual for commercially available callipers must also be updated. Manufacturers must determine and provide the following: category (Original, Generic or Hybrid); configuration (Type A, Type B or Type C); material (metal or plastic); lever class (first, second or third); jaw surface area (mm²); static downscale force (g) and pressure (g/mm²); dial type (scale or indicator); resolution and measurement range (mm); and weight without case (g). Some field calibration procedures have been proposed. The *Gauge-Block Test* and *Scale Test* are recommended to assess the accuracy (mm) and pressure (g/mm²) of skinfold callipers, respectively^(9)^. Despite this, repairing or replacing critical components, such as the jaws, spring, pivot and dial, remains challenging, particularly in clinical and field settings, as it requires technical expertise and specialised instruments. Consequently, manufacturers should be encouraged to provide ongoing, affordable maintenance services, preferably free of charge, to ensure the functionality, reliability and longevity of skinfold callipers^(9)^.

Researchers have described important systemic differences among skinfold callipers^(11,14,15,20,21)^. Cintra, Ripka and Heymsfield^(9)^ indicated in a scientific report that any original, generic or hybrid skinfold calliper, under favourable calibration conditions, can be used to assess body adiposity based on the comparison of skinfold thicknesses over time. However, based on mathematical prediction models and normative reference scales, the same skinfold calliper employed in the original studies should be used. Thus, the regression equations proposed by Durnin and Womersley^(22)^ and Jackson and Pollock^(23)^ to estimate body density and convert it into body fat percentage are important examples. They should be used based on skinfold thicknesses measured with original *Type A* and *Type B* skinfold callipers, respectively, or, when this is not possible, with their generic equivalents. A contrary approach results in significantly overestimated or underestimated relative and absolute values^(20,21)^, since the Harpenden^®^ calliper applies approximately three times more static downscale force than the Lange^™^ calliper (743 g *v*. 250 g, respectively) to the subcutaneous tissue, while the Slim Guide^®^ calliper applies 683 g, comparable to the force exerted by the Harpenden^®^ calliper^(9,11)^. This has a direct impact on skinfold thickness measurement^(9,11,15)^. Correction factors have been proposed as suitable alternatives to original and generic skinfold callipers^(21,24)^. When this is neglected by anthropometrists and researchers, the systematic bias produced by the *calliper-equation* conflict can affect resting energy expenditure estimated from fat-free mass derived from fat mass determined by skinfold thickness^(25,26)^. Indeed, this represents a relevant practical implication that future studies should directly address. Furthermore, inaccurate anthropometric measurements can also compromise the accuracy of body composition estimates. Machado *et al.* ^(27)^ observed significant variations between skinfold thicknesses at eight selected sites, obtained by anthropometrists with different levels of experience, resulting in substantial errors in the estimation and classification of total body adiposity. Therefore, standardised protocols, calibrated instruments and continued specialisation are critical factors in improving the skinfold technique and, consequently, data interpretation and health recommendations^(2,7,27)^.

Although the proposed organisational framework for skinfold callipers represents a significant conceptual advance, some limitations should be acknowledged. In particular, its practical application across different scenarios and contexts still depends on close cooperation between manufacturers, metrological institutions and scientific societies to establish technical standards based on critical physical-mechanical specificities. This article therefore urges manufacturers to clearly report the discussed characteristics of callipers, ensuring their proper classification and enabling the evaluation of their validity in anthropometric measurement. Likewise, researchers are encouraged to provide and disclose this information whenever appropriate. Finally, comparative studies between different instruments, conducted under standardised calibration conditions and involving diverse population samples, are essential to support their integration into international guidelines.

## Conclusion

This report proposed an innovative organisation of skinfold callipers into three categories (*Original*, *Generic* and *Hybrid*) and three configurations (*Type A*, *Type B* and *Type C*), based on physical-mechanical properties and characteristics, thus providing a systematic approach to their use and technical support for choosing the most appropriate calliper in different contexts of body adiposity assessment. Given its structured, integrative nature and its foundation in objective criteria, this proposal can therefore be referred to as *The Cintra Classification*. Finally, we also suggest that skinfold callipers be described in the literature based on their category, configuration, trade name and/or model, manufacturer and country, for example: *Original Type B skinfold calliper* (*Lange* ^*™*^ *, Beta Technology* ^*®*^).

## References

[1] Prado CM , Gonzalez MC , Norman K , et al. (2025) Methodological standards for body composition-an expert-endorsed guide for research and clinical applications: levels, models, and terminology. Am J Clin Nutr 122, 384–391. 40754386 10.1016/j.ajcnut.2025.05.022 PMC12405783

[2] Ripka WL , Cintra-Andrade JH & Ulbricht L (2022) A century of skinfolds for body composition estimation: what we learned? Rev Bras Cineantropom Desempenho Hum 24, e85412.

[3] Campa F , Coratella G , Petri C , et al. (2025) From fat to facts: anthropometric references and centile curves for sum of skinfolds and waist-to-hip ratio in 2507 adults. PLoS One 20, e0326111. 40569960 10.1371/journal.pone.0326111 PMC12200776

[4] Heymsfield SB (2024) Advances in body composition: a 100-year journey. Int J Obes 49, 177–181. 10.1038/s41366-024-01511-9 PMC11805704 38643327

[5] Serafini S , Charrier D , Izzicupo P , et al. (2025) Anthropometric-based predictive equations developed with multi-component models for estimating body composition in athletes. Eur J Appl Physiol 125, 595–610. 39641837 10.1007/s00421-024-05672-3

[6] Cerullo G , Franchi MV , Sampieri A , et al. (2025) Ultrasound-derived skinfolds in anthropometric predictive equations overestimate fat mass: a validation study using a four-component model. Nutrients 17, 1881. 40507151 10.3390/nu17111881 PMC12157048

[7] Cintra-Andrade JH , Brito FO , Freire-Correia MI , et al. (2023) Pinch size can affect the skinfold thickness measurement and interfere in the estimation and classification of body adiposity. Rev Bras Cineantropom Desempenho Hum 25, e90282.

[8] Campa F , Gobbo LA , Stagi S , et al. (2022) Bioelectrical impedance analysis *v.* reference methods in the assessment of body composition in athletes. Eur J Appl Physiol 122, 561–589. 35067750 10.1007/s00421-021-04879-y

[9] Cintra-Andrade JH , Ripka WL & Heymsfield SB (2023) Skinfold calipers: which instrument to use? J Nutr Sci 12, e82. 37528836 10.1017/jns.2023.58 PMC10388409

[10] Cerqueira MS , Amorim PRS , Encarnação IGA , et al. (2022) Equations based on anthropometric measurements for adipose tissue, body fat, or body density prediction in children and adolescents: a scoping review. Eat Weight Disord 27, 2321–2338. 35699918 10.1007/s40519-022-01405-7

[11] Schmidt PK & Carter JE (1990) Static and dynamic differences among five types of skinfold calipers. Hum Biol 62, 369–388. 2373507

[12] Edwards DA , Hammond WH , Healy MJ , et al. (1955) Design and accuracy of calipers for measuring subcutaneous tissue thickness. Br J Nutr 9, 133–143. 14389631 10.1079/bjn19550021

[13] Lange KO & Brozek J (1961) A new model of skinfold caliper. Am J Phys Anthrop 19, 98–99.

[14] Lohman TG , Pollock ML , Slaughter MH , et al. (1984) Methodological factors and the prediction of body fat in female athletes. Med Sci Sports Exerc 16, 92–96. 6708788

[15] Esparza-Ros F , Moreira AC , Vaquero-Cristóbal R , et al. (2022) Differences between four skinfold calipers in the assessment of adipose tissue in young adult healthy population. Nutrients 14, 2085. 35631225 10.3390/nu14102085 PMC9144069

[16] Esparza-Ros F , Vaquero-Cristóbal R & Marfell-Jones MJ (2019) International Standards for Anthropometric Assessment. Murcia: International Society for the Advancement of Kinanthropometry (ISAK).

[17] Jones PR , Marshall WA & Branson SJ (1979) Harpenden electronic read-out (HERO) skinfold calipers. Ann Hum Biol 6, 159–162. 475327 10.1080/03014467900003491

[18] Cintra-Andrade JH , Alves Martins JV , Freire-Correia MI , et al. (2025) Validation of portable ultrasonic stadiometers in adults. Clin Nutr ESPEN 68, 8–13. 40345653 10.1016/j.clnesp.2025.05.010

[19] Leão C , Clemente FM , Silva B , et al. (2023) Testing the concurrent validity and reliability of a Lipowise digital skinfold caliper to assess muscle mass in healthy young adults. Heliyon 9, e17569. 37408882 10.1016/j.heliyon.2023.e17569 PMC10319220

[20] Cyrino ES , Okano AH , Glaner MF , et al. (2003) Impact of the use of different skinfold calipers for the analysis of the body composition. Rev Bras Med Esporte 9, 150–153.

[21] Gruber JJ , Pollock ML , Graves JE , et al. (1990) Comparison of Harpenden and Lange calipers in predicting body composition. Res Q Exerc Sport 61, 184–190. 2094930 10.1080/02701367.1990.10608673

[22] Durnin JV & Womersley J (1974) Body fat assessed from total body density and its estimation from skinfold thickness: measurements on 481 men and women aged from 16 to 72 years. Br J Nutr 32, 77–97. 4843734 10.1079/bjn19740060

[23] Jackson AS & Pollock ML (1978) Generalized equations for predicting body density of men. Br J Nutr 40, 497–504. 718832 10.1079/bjn19780152

[24] Okano AH , Carvalho FO , Cyrino ES , et al. (2008) Use of the Cescorf skinfold caliper to estimate relative fatness from equations validated with the Lange caliper. Rev Educ Fís/UEM 19, 431–436.

[25] Cunningham JJ (1991) Body composition as a determinant of energy expenditure: a synthetic review and a proposed general prediction equation. Am J Clin Nutr 54, 963–969. 1957828 10.1093/ajcn/54.6.963

[26] Tinsley GM , Graybeal AJ & Moore ML (2019) Resting metabolic rate in muscular physique athletes: validity of existing methods and development of new prediction equations. Appl Physiol Nutr Metab 44, 397–406. 30240568 10.1139/apnm-2018-0412

[27] Machado DRL , Silva LSL , Vaquero-Cristóbal R , et al. (2025) Reliability of skinfold measurements and body fat prediction depends on the rater’s experience: a cross-sectional analysis comparing expert and novice anthropometrists. Sport Sci Health 21. 10.1007/s11332-025-01389-8

